# The paradox of relief: how decompression sets the stage for hydrocephalus—a retrospective inquiry into its clinical determinants

**DOI:** 10.1007/s00701-026-06865-9

**Published:** 2026-04-22

**Authors:** Muhammad Daniyal, Muhammad Samir Irfan Wasi, David Davies, Anna Podlasek

**Affiliations:** 1 Department of Neurosurgery, Queen Elizabeth Hospital Birmingham, Birmingham, UK; 2 Image Guided Therapy Research Facility, University of Dundee, Dundee, UK; 3 School of Medicine, University of St Andrews, St Andrews, UK; 4 Neurousurgery, University of Birmingham, Birmingham, UK

**Keywords:** Post-traumatic hydrocephalus, Decompressive craniectomy, Traumatic brain injury, Hygroma, Risk factors, Surveillance

## Abstract

**Background:**

Post-traumatic hydrocephalus (PTH) is a common complication following decompressive craniectomy for traumatic brain injury (TBI), occurring in 7–36% of patients. Despite its significant impact on recovery and long-term outcomes, the relative contribution of pre-operative, intra-operative, and post-operative factors to PTH development remains incompletely understood.

**Objective:**

To identify and quantify independent risk factors for PTH following decompressive craniectomy and develop a clinically applicable risk stratification framework.

**Methods:**

A retrospective cohort analysis of 142 consecutive patients who underwent decompressive craniectomy for TBI was conducted. The primary outcome was radiologically defined hydrocephalus. Pre-operative, intra-operative, and post-operative clinical and radiological variables were analysed using univariable and multivariable logistic regression. Model performance was assessed using area under the receiver operating characteristic curve (AUC), Hosmer–Lemeshow goodness-of-fit testing, and pseudo-R^2^ values.

**Results:**

Hydrocephalus developed in 43 patients (30.3%). Univariable analysis identified lower Glasgow Coma Scale (GCS) on arrival, post-operative interhemispheric hygroma, subdural hygroma, herniation, intraventricular haemorrhage, and pseudomeningocele as significant predictors (all P < 0.01). Intraoperative surgical parameters showed no association with PTH. The final multivariable model identified two independent predictors: lower GCS on arrival (OR 0.88, 95% CI 0.79–0.99; P = 0.032) and post-operative interhemispheric hygroma (OR 4.89, 95% CI 1.95–12.28; P = 0.0007). The model demonstrated strong discrimination (AUC = 0.807) and excellent calibration (Hosmer–Lemeshow P = 0.69), with overall classification accuracy of 81.0%. GCS score of ≤ 8 on arrival was significantly associated with increased hydrocephalus risk in binary analysis (*P* = 0.007), whereas the same threshold at intubation was not significant.

**Conclusions:**

Post-operative complications, particularly interhemispheric hygroma, are the strongest predictors of PTH following decompressive craniectomy, while surgical technique parameters show no significant association. These findings support the implementation of structured post-operative surveillance protocols to enable early detection and intervention in high-risk patients. A clinical risk assessment checklist incorporating these factors may improve prognostic accuracy and guide individualised management strategies.

## Introduction

Traumatic brain injury (TBI) is one of the leading causes of mortality worldwide. It affects approximately 69 million people annually, and a multifactorial aetiology engenders grave outcomes in these patients [5, 10]. Decompressive craniectomy is a lifesaving procedure performed routinely in patients with refractory raised intracranial pressure in TBI, aiming to reduce the mortality rate and improve outcomes [2, 15]. Post-traumatic hydrocephalus (PTH) is one of the most common complications in post-operative patients with decompressive craniectomy and is particularly notable due to its impact on recovery and quality of life [1, 14, 15, 20].

Development of hydrocephalus, characterised by abnormal cerebrospinal fluid (CSF) dynamics and ventriculomegaly, occurs in 7–36% of TBI patients post craniectomy, with rates varying widely in these reports due to differences in diagnostic criteria and patient populations [1, 5, 20]. It can lead to cognitive decline, gait disturbances, and incontinence, further complicating rehabilitation and long-term outcomes [1, 15].

Multiple hypotheses exist as to the mechanistic factors that lead to the development of hydrocephalus after decompressive surgery. This includes an ‘open box’ hypothesis, where intracranial pressure gradient and drive are removed. This may be potentially exacerbated by impaired CSF absorption by inflammation and fibrosis within the arachnoid villi, which potentiates the development of CSF accumulation in the absence of sufficient intracranial pressure to drive reabsorption. These can lead to the preferential dilatation of the ventricles and accompanying deformity of neurological structures.

It is essential to identify risk factors for this pathology to optimise the surgical approach to decompressive surgery and to minimise the risk of secondary hydrocephalus.

In our study, we aim to quantify the potential predisposing factors contributing to this mechanism.

## Methods

This is a retrospective cohort analysis of 142 consecutive patients who underwent decompressive craniectomy and imaging follow-up at University Hospitals Birmingham NHS Trust over the last 7 years. Patients with previous cranial surgeries not due to TBI, who died during initial admission, lost to follow-up within 6 months, bone flap replaced at the end of planned decompressive craniectomy, inadequate data (no initial CT scan) and previous shunts were excluded. We included patients of all ages, all severities of TBI and with a history of previous TBI. The demographics and clinical characteristics of patients undergoing decompressive craniectomy are shown in Table 1.

**Table 1:** Baseline demographic, clinical, radiological, intra-operative and post-operative characteristics of patients undergoing decompressive craniectomy (*N* = 142)

Variables	Number of patients (*N*)
Number of patients	142
Hydrocephalus	43 (30.3%)
No hydrocephalus	99 (69.7%)
Age (years)	39.80 ± 13.58
GCS on admission	8.89 ± 4.08
GCS after intubation	6.77 ± 3.33
**Pre-operative findings**	
Midline shift on CTH (mm)	8.86 ± 6.05
Subarachnoid haemorrhage	116 (81.7%)
Intraparenchymal haemorrhage	73 (51.4%)
Subdural hematoma	82 (57.7%)
Epidural hematoma	34 (23.9%)
Skull vault fracture	79 (55.6%)
Base of skull fracture	61 (43.0%)
Basal cisterns effaced	64 (45.1%)
Basal cistern haemorrhage	18 (12.7%)
Cerebral herniation	92 (64.8%)
Intraventricular haemorrhage	43 (30.3%)
Pre-operative ICP (mmHg)	25.45 ± 10.44*
**Post-operative findings**	
Intraventricular haemorrhage	29 (20.4%)
Post-operative ICP (mmHg)	18.39 ± 6.20
Post-operative midline shift (mm)	4.46 ± 3.55
Subdural hygroma	28 (19.7%)
Intra- axial hygroma	26 (18.3%)
Post-operative cerebral herniation	36 (25.4%)
Intracranial infection	19 (13.4%)
Sunken flap	27 (19.0%)
Syndrome of trephined	7 (5.0%)*

*Available in subset of patients. Values are presented as mean ± SD or number (%)

The primary outcome was the development of hydrocephalus, defined radiologically and coded as a binary variable (1 = hydrocephalus, 0 = no hydrocephalus). Pre-operative, intra-operative, and post-operative clinical and radiological variables were extracted from electronic records and imaging reports and reviewed manually. Interhemispheric hygroma was defined as a CSF-density collection within the interhemispheric fissure on CT imaging, attributed to traumatic arachnoid disruption and confirmed by neuroradiological reporting, while subdural hygroma was defined as an extra-axial CSF-density collection over the cerebral convexities without septations or haemorrhagic components.

Univariable logistic regression was performed for each predictor using MedCalc® (version 23.3.1). For each model, odds ratios (ORs) with 95% confidence intervals (CIs) were estimated. Model fit was assessed using − 2 Log Likelihood, the Hosmer–Lemeshow goodness-of-fit test, and pseudo-R^2^ (Cox–Snell, Nagelkerke). Discrimination was quantified using the area under the receiver operating characteristic curve (AUC). Classification tables were generated using a probability threshold of 0.5. No imputation was performed; analyses used complete case data for each variable. Predictors reaching P < 0.10 in univariable analysis were taken forward for multivariable modelling. Multicollinearity was assessed using Variance Inflation Factors (VIF).

## Results

### Study population

A total of 142 patients were included; 43 (30.3%) developed hydrocephalus and 99 (69.7%) did not.

### Pre-operative predictors (Table 2)

**Table 2:** Univariate analysis of preoperative and post operative factors associated with hydrocephalus (*N* = 142)

Variable	Odds ratio	95% CI	*P* value
**Preoperative**			
Age	1	0.97–1.02	0.8478
GCS on admission	0.85	0.77–0.94	0.0011
GCS after intubation	0.85	0.76–0.96	0.0094
SAH	2.79	0.90–8.65	0.0763
IPH	1	0.43–2.33	0.9976
SDH	0.39	0.13–1.19	0.0995
EDH	1.39	0.53–3.60	0.5007
Skull vault fracture	1.99	0.83–4.80	0.1231
BOS fracture	1.36	0.63–2.92	0.4323
Basal cisterns effaced	1.38	0.67–2.83	0.3793
Basal cistern blood	1.73	0.52–5.79	0.3743
Cerebral herniation	1.02	0.48–2.16	0.957
Intraventricular haemorrhage (IVH)	1.66	0.70–3.92	0.2499
Midline shift on CT head (mm)	1.03	0.96–1.11	0.3521
Pre-op ICP	0.96	0.88–1.04	0.3025
**Post-operative**			
Post-op new IVH	3.87	1.49–10.07	0.0055
Post-op MLS (mm)	0.93	0.82–1.06	0.2764
Pseudomeningocele	3.11	1.46–6.65	0.0034
Post-op subdural hygroma	6.30	2.69–14.76	< 0.0001
Interhemispheral hygroma	8.36	3.61–19.32	< 0.0001
Post-op brain herniation	5.24	2.43–11.33	< 0.0001
Intracranial infection	4.74	2.01–11.19	0.0004
Sunken flap	1.05	0.43–2.53	0.9212
Syndrome of trephined	3.25	0.69–15.19	0.1345

Most pre-operative variables were not significantly associated with hydrocephalus. Measures of baseline neurological status were the only factors demonstrating statistically significant associations:

- **Lower GCS on arrival** was associated with increased risk of hydrocephalus (OR 0.84, 95% CI 0.76–0.93; P = 0.0005), with fair model discrimination (AUC 0.689).
- **Lower GCS at intubation** also predicted hydrocephalus (OR 0.85, 95% CI 0.76–0.96; P = 0.009), though with lower discriminative performance (AUC 0.639).

Other pre-operative variables—including age, craniotomy status, presence of effaced basal cisterns, IVH, midline shift, herniation subtypes, DM, HTN, stroke, TBI, and psychiatric history—showed no statistically significant association with hydrocephalus (all P > 0.10). Effect sizes were small and confidence intervals wide, and all models demonstrated limited discriminatory ability (AUC ~ 0.50–0.56).

### Intra-operative predictors

None of the intra-operative variables assessed—C measurement, distance from midline, falx division, laterality, SA measurement, or intra-operative size—were significantly associated with hydrocephalus (all P > 0.15). Odds ratios were close to unity, with narrow confidence intervals spanning no effect. All models showed poor discrimination (AUC 0.50–0.54).

### Post-operative predictors (Table 2)

Post-operative factors demonstrated the strongest and most clinically meaningful associations with hydrocephalus:

- **Intra-cranial hygromas** were strongly associated:
  - ◌ Interhemispheric hygroma: OR 8.36 (95% CI 3.61–19.32; *P* < 0.0001; AUC 0.713)
  - ◌ Subdural hygroma: OR 6.30 (95% CI 2.69–14.76; *P* < 0.0001; AUC 0.672)
- **Post-operative herniation** increased hydrocephalus risk (OR 5.24, 95% CI 2.43–11.33; *P* < 0.0001; AUC 0.694).
- **Post-operative IVH** was also significant (OR 3.87, 95% CI 1.49–10.07; *P* = 0.005), though with modest discrimination (AUC 0.594).
- **Pseudomeningocele** showed a moderate association (OR 3.15, 95% CI 1.48–6.73; *P* = 0.003; AUC 0.628).

Trephined bone flap status and sunken flap appearance were not associated with hydrocephalus.

### Multivariable modelling

#### Combined pre-operative model (Table 3)

**Table 3:** Multivariable model for preoperative and post operative predictors of hydrocephalus (*N* = 142)

Variable	Coefficient	Std. Error	Adjusted OR	95% CI	*P* value
**Preoperative**					
GCS on admission	−0.112	0.0708	0.89	0.78–1.03	0.114
GCS after intubation	−0.1012	0.0917	0.90	0.76–1.08	0.270
SAH	0.8936	0.6082	2.44	0.74–8.05	0.142
SDH	−1.2247	0.6571	0.29	0.08–1.07	0.064
CVST	0.4981	0.5139	1.65	0.60–4.51	0.332
**Post-Operative**					
Post-op IVH	0.80	0.64	2.23	0.64–7.82	0.210
Pseudomeningocele	0.55	0.47	1.74	0.69–4.38	0.241
Post-op subdural hygroma	0.66	0.56	1.93	0.64–5.81	0.244
Interhemispheric hygroma	1.22	0.56	3.39	1.12–10.24	0.031
Post-op brain herniation	0.72	0.53	2.06	0.73–5.77	0.171
Intracranial infection	0.70	0.57	2.02	0.66–6.21	0.221

A multivariable model incorporating GCS on arrival and GCS at intubation demonstrated modest explanatory power (R^2^ = 0.0915). Only **lower GCS on arrival** remained an independent predictor (β =  − 0.031, P = 0.0125), with acceptable collinearity diagnostics (VIF = 1.79).

#### Combined post-operative model (Table 3)

When all significant post-operative variables were entered simultaneously (CSF diversion, herniation, interhemispheric hygroma, subdural hygroma, IVH, pseudomeningocele), the model showed stronger explanatory ability (R^2^ = 0.3297, adjusted R^2^ = 0.2999). Independent associations persisted for interhemispheric hygroma (β = 0.218, P = 0.0265).

Other predictors lost statistical significance after adjustment, and collinearity remained low (VIF 1.08–1.73). Post-operative intracranial infection occurred in 19 patients (13.4%) and was significantly associated with hydrocephalus on univariable analysis; however, this did not remain significant in the multivariable model.

#### Final multivariable logistic regression model

A combined logistic regression including all variables that remained significant after staged modelling identified two independent predictors of hydrocephalus (Table 4):

- **Lower GCS on arrival** (OR 0.90, 95% CI 0.81–0.99; *P* = 0.048)
- **Post-operative interhemispheric hygroma** (OR 4.81, 95% CI 1.92–12.06; *P* = 0.0007)
- **Post operative new Intra-ventricular hemorrhage** (OR 3.01, 95% CI 1.15–7.88; *P* = 0.025)

**Table 4:** Final multivariable logistic regression model for hydrocephalus (*N* = 142)

Variable	Coefficient	Std. Error	Adjusted OR	95% CI	*P* value
GCS on admission	− 0.106	0.054	0.90	0.81–0.99	0.048
Post-op new IVH	1.10	0.49	3.01	1.15–7.88	0.025
Intra- axial hygroma	1.57	0.47	4.81	1.92–12.06	0.0007

Final model performance: − 2LL = 128.39; Cox–Snell R2 = 0.276; Nagelkerke R2 = 0.390; AUC = 0.824; HL P = 0.564

Multicollinearity: VIF range 1.00–1.50

The final model demonstrated good overall performance, with Nagelkerke R^2^ = 0.383, excellent calibration (Hosmer–Lemeshow *P* = 0.69), and AUC = 0.807, indicating strong discrimination. Classification accuracy improved to **81.0%**, particularly through improved identification of hydrocephalus cases (53.5% correctly predicted vs 0% in univariable models).

Lower GCS on arrival, analysed as a continuous variable, was significantly associated with an increased risk of hydrocephalus (OR 0.84, 95% CI 0.76–0.93; *p* = 0.0005), with fair model discrimination (AUC 0.689). Lower GCS at the time of intubation, when analysed continuously, was also a significant predictor of hydrocephalus (OR 0.85, 95% CI 0.76–0.96; *p* = 0.009), although with lower discriminative performance (AUC 0.639). When analysed as a binary variable, a GCS score of ≤ 8 on arrival was significantly associated with increased hydrocephalus risk (OR 2.81, 95% CI 1.33–5.96; *p* = 0.007), with fair discrimination (AUC 0.625). However, a GCS score of ≤ 8 at intubation was not significantly associated with hydrocephalus (OR 1.98, 95% CI 0.85–4.59; *p* = 0.134) and demonstrated poor model discrimination (AUC 0.567).

## Discussion

Hydrocephalus post-decompressive craniectomy is a well reported phenomenon with studies reporting the incidence of 7–36% [15]. Multiple risk factors have been identified, including advanced age, lower Glasgow Coma Scale (GCS) at admission, subarachnoid or intraventricular haemorrhage, subdural or interhemispheric hygroma, post-traumatic cerebral infarction, and proximity of the craniectomy margin to the midline (< 21–25 mm) [1, 3, 8, 9, 14, 15, 17, 18, 20]. The presence of subdural or interhemispheric hygroma is a strong independent predictor of PTH, with meta-analyses showing a significant association (hazard ratio up to 7.1) [13, 14, 17, 20]. 

The pathophysiology of PTH post-decompressive craniectomy is multifactorial, involving CSF malabsorption due to blood products, altered CSF flow dynamics from the craniectomy defect and secondary brain injury phenomena such as infarction or progression of contusion [15, 20]. While surgical technique parameters (e.g., craniectomy size) are less consistently linked to PTH, a margin too close to the midline is a modifiable risk factor [1, 3, 18]. 

### Key findings and risk stratifications

Our study identified three independent predictors of post-traumatic hydrocephalus in a cohort of 142 patients who underwent decompressive craniectomy. The final multivariable model demonstrated that lower GCS on arrival (OR 0.88, 95% CI 0.79–0.99) and post-operative interhemispheric hygroma (OR 4.89, 95% CI 1.95–12.28) were the strongest independent risk factors, with excellent overall model performance (AUC = 0.807). These findings align with previous literature identifying lower GCS scores and hygroma formation as significant predictors [15, 20]. Advanced age, which has been reported as a significant risk factor in multiple studies [15, 20], did not reach statistical significance in our study. This discrepancy may reflect differences in patient populations or the predominance of post-operative factors in our model.

**Fig. 1:**
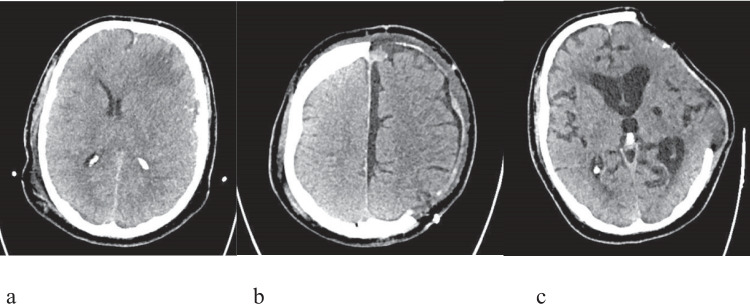
Illustrative Case- 64 years old gentleman presented after a fall downstairs with GCS of 3/15 and was intubated at the scene. Initial CT Head (**a**) showed multifocal parenchymal and traumatic subarachnoid haemorrhage, thin acute subdural hematoma (4 mm) and contusions – primarily left-sided. Decompressive craniectomy was done, and a postoperative scan on day 2 (**b**) showed interhemispheric and subdural hygroma. He made a slow recovery to GCS 7 t but developed hydrocephalus on postoperative day 12 (**c**). This was managed with a lumbar drain, which was subsequently challenged and removed before discharge to the rehabilitation unit

Our results emphasise the critical importance of post-operative radiological findings over pre-operative and intra-operative surgical parameters. Intra-operative variables such as craniectomy size, distance from midline, and falx division showed no significant association with PTH development in our study. This contrasts with some reports suggesting that large craniectomy area and proximity to the midline are risk factors [6, 11]. However, our findings are consistent with recent systematic reviews highlighting the inconsistent relationship between surgical technique and PTH [7]. An illustrative case demonstrating the development of post-traumatic hydrocephalus following decompressive craniectomy is shown in Fig. 1.

### Proposed risk assessment checklist

Based on our findings and existing literature, we propose a risk assessment checklist for PTH following decompressive craniectomy.

*Pre-operative Factors:*

- Glasgow Coma Scale score on arrival (particularly GCS ≤ 8, which showed a significant association in binary analysis) [20].
- Presence of intraventricular haemorrhage [20].
- Subarachnoid haemorrhage in basal cisterns [15].
- Post-traumatic ischemic infarcts [15].

*Post-operative Factors (High Priority):*

- Development of subdural or interhemispheric hygroma [9, 11].
- Post-operative interhemispheric hygroma
- Transcalvarial brain herniation [6, 15].
- Post-operative intraventricular haemorrhage.
- Progression of contusion [15].
- Subdural effusion [9].

*Additional Considerations*

- Post-operative meningitis [20].
- Post-traumatic cerebral infarction [20].

Patients presenting with multiple risk factors, particularly post-operative hygroma development and low admission GCS, should be flagged for intensive surveillance.

### Clinical implications and surveillance strategies

In our study, the strong association between post-operative findings and PTH development has important clinical implications for surveillance protocols. Given that post-operative interhemispheric hygroma demonstrated the highest odds ratio (OR 4.89) in our final model, we recommend structured post-operative imaging surveillance, particularly in high-risk patients. Studies have shown that PTH can develop anywhere from within the first week to several months post-injury, with the first 50 days being the most critical period  [14]. In our analysis, post-operative factors emerged as the dominant predictors, suggesting that the pathophysiological cascade leading to PTH is largely established in the immediate post-operative period.

We propose the following surveillance strategy:

1. *Early post-operative imaging (days 5–10):* To identify hygroma formation, herniation, and evolving haemorrhagic changes.
2. *Immediate follow-up (2–4 weeks):* Serial imaging for patients with identified risk factors, particularly those with hygroma or herniation [9].
3. *Extended surveillance (3–6 months):* Clinical assessment and imaging for symptomatic patients or those with persistent ventriculomegaly [19].

Post-operative clinical review should include neurological examination focusing on signs of raised intracranial pressure, cognitive decline, gait disturbances, and urinary incontinence. Early detection of symptomatic PTH allows for timely intervention with CSF diversion procedures, which may improve functional outcomes [4, 12]. Studies have demonstrated that patients with low-pressure hydrocephalus symptoms show the best response to shunt treatment, with improvement rates of approximately 66% [8]. However, it is crucial to distinguish between post-traumatic ventriculomegaly and true hydrocephalus requiring surgical intervention, as vegetative patients show minimal shunt response [12].

The timing of cranioplasty also warrants consideration in the context of PTH risk. While early cranioplasty (< 90 days) has been associated with reduced rates of certain complications, delayed cranioplasty increases the risk of hygroma formation and potentially PTH [7]. Some studies suggest that simultaneous cranioplasty and ventriculoperitoneal shunt procedures can be performed safely in selected patients, though this remains a topic of ongoing investigations [16].

### Study limitations

Our study has several limitations that should be acknowledged. First, as a single-centre retrospective study, our findings may not be generalizable to all patient populations or healthcare settings. Selection bias may have been introduced through our exclusion criteria, particularly the exclusion of patients who died during initial admission, which may have removed the most severely injured patients from analysis.

Second, our definition of hydrocephalus was based on radiological criteria without systematic incorporation of clinical symptoms or standardised diagnostic assessments such as invasive pressure monitoring or CSF tap tests. This may have led to inclusion of asymptomatic ventriculomegaly cases that would not have required intervention [19]. The distinction between post-traumatic ventriculomegaly and true symptomatic hydrocephalus remains challenging, and our study did not systematically differentiate these entities.

Third, we did not capture data on certain variables that have been identified as risk factors in other studies, including specific details about postoperative meningitis [20], the exact timing and volume of hygroma development, or quantitative measures of brain herniation volume [6]. The absence of standardised follow-up imaging protocols may have introduced detection bias, as patients with more concerning clinical courses may have undergone more frequent imaging.

Fourth, our sample size of 142 patients, while reasonable for a single-centre study, may have limited our statistical power to detect associations with less common risk factors. The relatively low event rate (43 patients with hydrocephalus) may explain why some previously reported risk factors, such as advanced age, did not reach statistical significance in our multivariable model.

Finally, we did not assess long-term functional outcomes or the impact of PTH on rehabilitation and quality of life. Because cranioplasty timing was not systematically captured for all patients, it could not be included in the statistical analysis. Long term outcomes, however, would not benefit in concluding the aim of this study as it focuses on predictors of hydrocephalus.

Future prospective studies with standardised diagnostic criteria, systematic surveillance protocols, and long-term outcome assessments are needed to validate our findings and refine risk prediction models. Multicentre collaboration would enhance generalizability and allow for the development of robust clinical decision support tools [6].

Despite these limitations, our study provides valuable insights into the relative importance of different risk factors and highlights the critical role of post-operative surveillance in identifying patients at high risk for PTH development.

### Clinical significance

It is important to emphasize that the development of PTH is associated with poorer neurological outcomes, increased disability, and prolonged rehabilitation [4, 9, 15, 20]. Shunt-dependent patients generally experience worse functional outcomes compared to those without PTH, with shunt complications representing a strong independent predictor of unfavourable outcomes [4]. Early diagnosis and intervention, including shunt placement when indicated, can improve functional recovery, though careful patient selection is essential to optimize outcomes [4, 12]. The implementation of systematic risk assessment and surveillance protocols, as proposed in this study, may facilitate earlier detection and intervention, potentially improving long-term outcomes for this vulnerable patient at high risk for PTH development.

## Conclusion

Post-traumatic hydrocephalus following decompressive craniectomy represents a significant and predictable complication whose development is largely determined by post-operative factors rather than mechanism of injury or surgical technique. The implementation of systematic risk assessment and surveillance protocols, guided by the admission GCS, identification of post-operative hygroma formation and other high-risk features, may facilitate earlier detection and intervention in this vulnerable population. By transforming the paradox of relief into an opportunity for proactive management, neurosurgical teams can potentially mitigate one of the most consequential complications of decompressive craniectomy and improve outcomes for patients navigating the complex trajectory of severe traumatic brain injury recovery.

## Data Availability

The datasets generated and/or analysed during the current study are not publicly available due to NHS information governance and patient confidentiality requirements but may be available from the corresponding author on reasonable request and with appropriate approvals.

## References

[1] Bagherzadeh S, Bahari L, Roohollahi F (2025) Post-craniectomy hydrocephalus in adult traumatic brain injury patients: a systematic review and meta-analysis of risk factors and outcome. Neurosurg Rev 48(1):72. 10.1007/s10143-025-03232-7 39841279 10.1007/s10143-025-03232-7

[2] de Abreu SDF, de Almeida BFA, de Macedo VG, Gomes LN, de Figueirêdo GF, Nogueira APF (2025) Evaluation of postoperative outcomes following decompressive craniectomy in patients with intracranial hypertension due to severe traumatic brain injury. Braz J Implantol Health Sci 7(1):921–934. 10.36557/2674-8169.2025v7n1p921-934

[3] De Bonis P, Pompucci A, Mangiola A, Rigante L, Anile C (2010) Post-traumatic hydrocephalus after decompressive craniectomy: an underestimated risk factor. J Neurotrauma 27(11):1965–1970 20812777 10.1089/neu.2010.1425

[4] Deng H, Goldschmidt E, Nwachuku EL et al (2021) Hydrocephalus and cerebrospinal fluid analysis following severe traumatic brain injury: evaluation of a prospective cohort. Neurol Int 13(4):527–534. 10.3390/neurolint13040052 34698266 10.3390/neurolint13040052 PMC8544497

[5] Dewan MC, Rattani A, Gupta S et al (2018) Estimating the global incidence of traumatic brain injury. J Neurosurg 130(4):1080–1097. 10.3171/2017.10.JNS17352 29701556 10.3171/2017.10.JNS17352

[6] Gao Z, Zhang G, Xu C, Zhu Z, Jiang H (2025) Analysis of hydrocephalus after decompressive craniectomy for traumatic brain injury. Pak J Med Sci 41(8):11324. 10.12669/pjms.41.8.11324 10.12669/pjms.41.8.11324 PMC12444121 40980363

[7] Hannah E, Zyck S, Hazama A, Krishnamurthy S (2022) Scoping review of the risk factors and time frame for development of post-traumatic hydrocephalus. Rev Neurosci 33(2):133–146. 10.1515/revneuro-2021-0043 34144640 10.1515/revneuro-2021-0043

[8] Honeybul S, Ho KM (2012) Incidence and risk factors for post-traumatic hydrocephalus following decompressive craniectomy for intractable intracranial hypertension and evacuation of mass lesions. J Neurotrauma 29(10):1872–1878 22583391 10.1089/neu.2012.2356

[9] Hu Q, Di G, Shao X, Zhou W, Jiang X (2018) Predictors associated with post-traumatic hydrocephalus in patients with head injury undergoing unilateral decompressive craniectomy. Front Neurol 9:234 29867743 10.3389/fneur.2018.00337 PMC5960668

[10] James SL, Theadom A, Ellenbogen RG et al (2019) Global, regional, and national burden of traumatic brain injury and spinal cord injury, 1990–2016: a systematic analysis for the global burden of disease study 2016. Lancet Neurol 18(1):56–87. 10.1016/S1474-4422(18)30415-0 30497965 10.1016/S1474-4422(18)30415-0 PMC6291456

[11] Ki HJ, Lee HJ, Lee H, Yi JS, Yang JH, Lee I (2015) The risk factors for hydrocephalus and subdural hygroma after decompressive craniectomy in head injured patients. J Korean Neurosurg Soc 58(3):254–261. 10.3340/jkns.2015.58.3.254 26539270 10.3340/jkns.2015.58.3.254 PMC4630358

[12] Kowalski RG, Weintraub AH, Rubin BA, Gerber DJ, Olsen AS (2019) Impact of timing of ventriculoperitoneal shunt placement on outcome in posttraumatic hydrocephalus. J Neurosurg. 10.3171/2017.7.jns17555 29473779 10.3171/2017.7.JNS17555

[13] Lu VM, Carlstrom LP, Perry A et al (2021) Prognostic significance of subdural hygroma for post-traumatic hydrocephalus after decompressive craniectomy in the traumatic brain injury setting: a systematic review and meta-analysis. Neurosurg Rev 44(1):129–138 31845199 10.1007/s10143-019-01223-z

[14] Nasi D, Gladi M, Di Rienzo A et al (2018) Risk factors for post-traumatic hydrocephalus following decompressive craniectomy. Acta Neurochir (Wien) 160(9):1691–1698. 10.1007/s00701-018-3639-0 30054725 10.1007/s00701-018-3639-0

[15] Romualdo SMF, Juratli TA, Eyüpoglu I et al (2025) Post-traumatic hydrocephalus after decompressive craniectomy: a multidimensional analysis of clinical, radiological, and surgical risk factors. Neurosurg Rev 48(1):523. 10.1007/s10143-025-03673-0 40542880 10.1007/s10143-025-03673-0 PMC12182525

[16] Ting CH, Lu CW, Lan CW, Lee TC, Hsu SW, Su TM (2020) Simultaneous cranioplasty and ventriculoperitoneal shunt placement in patients with traumatic brain injury undergoing unilateral decompressive craniectomy. J Clin Neurosci 79:45–50. 10.1016/j.jocn.2020.07.015 33070916 10.1016/j.jocn.2020.07.015

[17] Vedantam A, Yamal JM, Hwang H, Robertson C, Gopinath S (2017) Factors associated with shunt-dependent hydrocephalus after decompressive craniectomy for traumatic brain injury. J Neurosurg 128(5):1547–1552 28621627 10.3171/2017.1.JNS162721

[18] Vinayaka GJ, Sharmad MS, Peethambaran AK, Kutty RK (2024) Posttraumatic hydrocephalus following Decompressive Craniectomy in Traumatic Brain Injury: proportion and risk factors. Indian J Neurotrauma (efirst). 10.1055/s-0044-1782608

[19] Wettervik T, Lewén A, Enblad P (2022) Post-traumatic hydrocephalus – incidence, risk factors, treatment, and clinical outcome. Br J Neurosurg 36(3):400–406. 10.1080/02688697.2021.1967289 34414834 10.1080/02688697.2021.1967289

[20] Yadav V, Sahu A, Pandey N et al (2024) A comprehensive study of risk factors predicting hydrocephalus following decompressive craniectomy in traumatic brain injuries. Egypt J Neurosurg 39(1):57. 10.1186/s41984-024-00323-3

